# Investigation of the Interaction between *Aloe vera* Anthraquinone Metabolites and *c-Myc* and *C-Kit* G-Quadruplex DNA Structures

**DOI:** 10.3390/ijms232416018

**Published:** 2022-12-16

**Authors:** Sabrina Dallavalle, Roberto Artali, Salvatore Princiotto, Loana Musso, Gigliola Borgonovo, Stefania Mazzini

**Affiliations:** 1 Department of Food, Environmental and Nutritional Sciences (DEFENS), University of Milan (Università degli Studi di Milano), 20133 Milan, Italy; 2 National Institute of Fundamental Studies, Kandy 20000, Sri Lanka; 3 Scientia Advice di Roberto Artali, 20832 Desio, Italy

**Keywords:** G-quadruplex, anthraquinones, *Aloe vera*, NMR spectroscopy, molecular modeling

## Abstract

G-quadruplexes are nucleotide sequences present in the promoter region of numerous oncogenes, having a key role in the suppression of gene transcription. Recently, the binding of anthraquinones from *Aloe vera* to G-quadruplex structures has been studied through various physico-chemical techniques. Intrigued by the reported results, we investigated the affinity of aloe emodin, aloe emodin-8-glucoside, and aloin to selected G-quadruplex nucleotide sequences by NMR spectroscopy. The structural determinants for the formation of the ligand/nucleotide complexes were elucidated and a model of the interactions between the tested compounds and *C-Kit* and *c-Myc* G-quadruplex DNA structures was built by integrated NMR and molecular modeling studies. Overall, the obtained results confirmed and implemented the previously reported findings, pointing out the complementarity of the different approaches and their contribution to a more detailed overview of the ligand/nucleotide complex formation. Furthermore, the proposed models of interaction could pave the way to the design of new nature-derived compounds endowed with increased G-quadruplex stabilizing activity.

## 1. Introduction

Among the various chemical classes of natural products, anthraquinones are characterized by a wide structural diversity and relevant biological activities [1,2,3]. Anthraquinones are mostly present in the families of Fabaceae (Cassia), Liliaceae (Aloe), Polygonaceae (Rumex), Rhamnaceae (Rhamnus), Rubiaceae (Asperula, Gallium, Rubia), and Scrophulariaceae [4].

**Figure 1:**
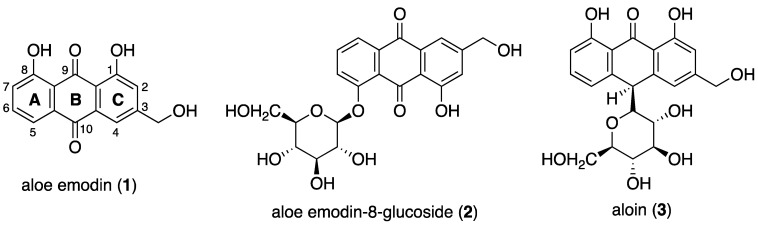
Structures of the main *A*. *vera* anthraquinones aloe emodin (**1**), aloe emodin-8-glucoside (**2**) and aloin (**3**).

Anthraquinone-based compounds such as daunomycin, doxorubicin, and mitoxantrone are used as chemotherapeutic agents due to their ability of targeting DNA [5,6]. Many other anthraquinones have been reported to have anticancer potential. Among them, *Aloe vera*-extracted anthraquinones such as aloe emodin (**1**), aloe emodin-8-glucoside (**2**), and aloin (**3**) have recently attracted great interest [7] (Figure 1).

Aloe emodin (**1**), which is one of the major Aloe active compounds, is known to inhibit cell proliferation in tumor cells [8,9]. Several reports suggest that aloe emodin has antiproliferative and antitumor activity in human hepatoma, lung carcinoma, and human gastric carcinoma, also having high specificity for neuroectodermal tumors [8,10,11]. Aloe emodin-8-glucoside (**2**) has shown antidiabetic activity in the treatment of insulin resistance [12], whereas the anthraquinone *C*-glycoside aloin (**3**) has a variety of pharmacological activities and potential chemotherapeutic properties [13,14,15].

In recent years we have focused our research activity on the investigation of small molecules working as G-quadruplex DNA stabilizers [16,17,18,19,20,21]. G-quadruplexes (G4) are stable structures found in guanine-rich regions of DNA. Four guanine bases can associate through Hoogsteen hydrogen bonding to form a square planar structure called guanine tetrad, with two or more guanine tetrads (from G-tracts, continuous runs of guanine) stacking on top of each other to form a G-quadruplex [22].

The widespread presence of G-quadruplexes in the human genome, including telomeres as well as the promoter region of numerous oncogenes, clearly indicates their exclusive role in gene regulation. As a consequence, selective targeting by small molecules can potentially result in stabilization of G-quadruplex structures and suppression of oncogene transcription [23]. The presence of the planar anthraquinone moiety in *Aloe vera* extracted compounds makes them attractive candidates in the search of new G-quadruplex stabilizers.

In a recent paper, Das and Dutta investigated the binding interaction of the three above reported anthraquinone derivatives with a variety of G-quadruplex sequences (*C-Kit*, *c-Myc*, HUMTEL, BCL-2, KRAS, and VEGF), by using different physico-chemical techniques such as absorption spectral titration, fluorescence spectral titration, dye displacement, ferrocyanide quenching assay, and CD and DSC thermogram studies [24]. In particular, aloe emodin **1** and aloe emodin-8-glucoside **2** emerged as potent G-quadruplex binders mostly in the case of *C-Kit* and *c-Myc* sequences, with a binding affinity higher than their duplex DNA binding ability, which has been previously determined by the same group [7,25]. Aloe emodin **1** showed the highest binding affinity (K = 10^5^ M^−1^) compared to aloe emodin-8-glucoside **2** (K = 10^4^ M^−1^) with all the considered quadruplex sequences. Conversely, aloin **3** (K = 10^3^ M^−1^) showed very poor G-quadruplex binding ability in comparison with the congeners **1** and **2**. The melting temperatures obtained for quadruplex DNA were 62.4 °C for *C-Kit* and 75.8 °C for *c-Myc*. **1** and its *O*-glucoside **2**, after complexation with *C-Kit* and *c-Myc*, increased the melting temperature by 4 °C and 2 °C, respectively. For its part, aloin did not show any stabilizing effect on the thermal melting of the quadruplex sequences [24].

Intrigued by these exciting results, we were prompted to deepen the study of the reported three anthraquinones, e.g., aloe-emodin, aloe-emodin-8-glucoside, and aloin. In particular, our efforts were directed to the investigation of their interaction with G-quadruplex structures by NMR spectroscopy, in order to elucidate the geometry of the complexes and the structural determinants of the interaction. Models of the complexes between the tested compounds and *C-Kit* and *c-Myc* structures were created as well. To the best of our knowledge, no example of such an investigation on natural products from the *A. vera* compound has been reported in the literature to date. 

Our results were in full agreement with those reported from Das and Dutta, confirming that the two approaches are robust and complementary and could be helpful in the design of new nature-inspired G-quadruplex stabilizers.

## 2. Results and Discussion

### 2.1. Interaction of Aloe Anthraquinones with c-kit21T12T21 and Pu22T14T23

**Scheme 1:**
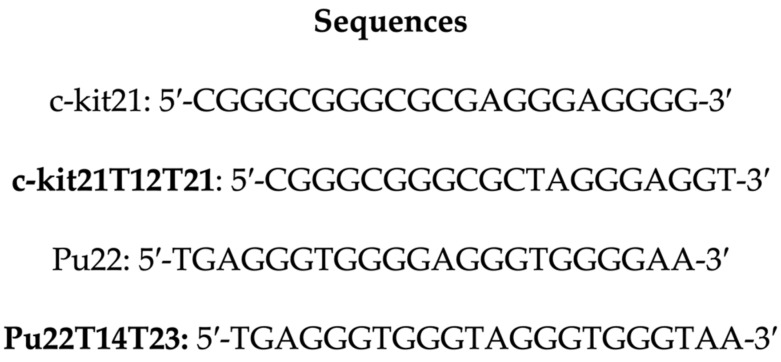
Schematic representation of the nucleotidic sequences in c-kit21 and Pu22 and their mutated counterparts.

The investigation was carried out by using c-kit21T12T21 and Pu22T14T23, which are sequences with two mutations at positions 12 and 21 of *C-Kit* and at positions 14 and 23 of *c-Myc* Pu22, with respect to the native sequences (Scheme 1).

Several authors have used these modified oligonucleotides to study the binding properties of G-quadruplex binding drugs by NMR, as the wild sequence gave poorly resolved proton NMR spectra [26,27,28]. The mutations allow the formation, for both the oligonucleotides, of a single monomeric intramolecular parallel G-quadruplex structure similar to the native forms [29,30,31]. As reported, 12 narrow and well-resolved imino protons are present in the ^1^H NMR spectrum of both oligonucleotides, which is consistent with the formation of G-quadruplexes with three G-quartets.

The ^1^H NMR spectra of c-kit21T12T21 and Pu22T14T23 without ligands have been attributed following the assignment already reported in literature [30,32]. Increasing amounts of ligands were added to the oligonucleotide solution. The assignment of the imino NH resonances of the guanines in all complexes with aloe derivatives was performed, starting from the 1D titration spectra and by sequential NH-NH NOE interactions carried out by NOESY experiments. Consequently, the assignments of the aromatic protons of the ligand in the complexes were possible by observing the inter-residue NOE interactions with the NH imino protons, which define the patterns of the three tetrads for both G-quadruplexes. The identification of the cytidine aromatic protons was based on TOCSY experiments, as the vicinal H6 and H5 protons displayed very strong signals. The thymine protons were identified through the methyl resonances, lying upfield. Selected chemical shifts for the considered complexes are reported in Tables S1–S5. The major problem during the titration experiments of aloe emodin (**1**) with both G-quadruplex sequences was the low water solubility of these compounds at the concentration required for the NMR experiments, which hampered the investigation at high drug/DNA ratios. The presence of the sugar moiety in aloe emodin-8-glucoside (**2**) increased its water solubility in comparison with aloe emodin (**1**). Moreover, the glucose ring increased the interactions with the quadruplex structure, being involved in the formation of non-covalent bonds (see the modeling section).

#### 2.1.1. Interaction of Aloe Emodin (**1**) and Aloe Emodin-8-glucoside (**2**) with c-kit21T12T21

The oligonucleotide c-kit21T12T21 displayed a high-quality NMR spectrum. In both complexes the NH imino protons chemical shifts, remaining in a region ranging from 10.5 to 12.0 ppm, indicated that the G-quadruplex structure was conserved after the interaction with the ligands.

**Figure 2:**
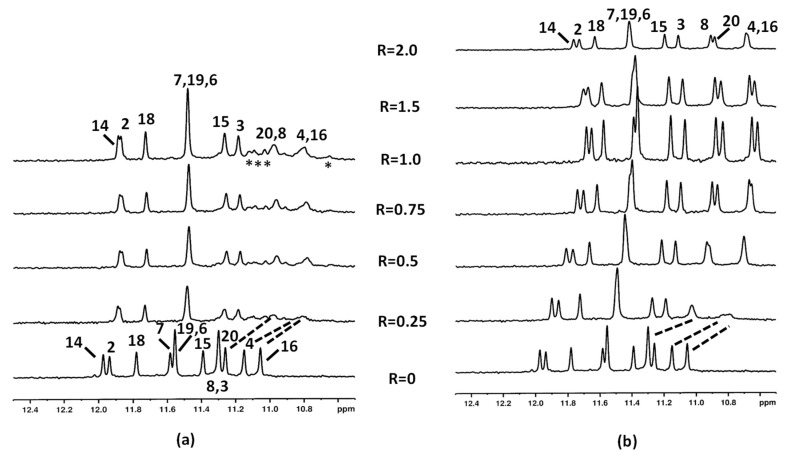
Imino protons region of the ^1^H NMR titration spectra of c-kit21T12T21 with (**a**) aloe emodin (**1**) and (**b**) aloe emodin-8-glucoside (**2**) (R = 2.0 after two hours of the addition). The numbers indicate the assignation to the corresponding imino protons, the dotted lines highlight the change in chemical shifts. The asterisks (*) mark the presence of new signals between 11.2 and 10.6 ppm. Spectra were acquired at 25 °C in 90% H_2_O and 10% D_2_O, 20 mM KCl, and 5 mM K-phosphate buffer, pH 6.9.

Following the imino proton signals during the titration experiments with aloe emodin (**1**) and aloe emodin-8-glucoside (**2**), important line broadening and an upfield chemical shift variation of the guanines G4, G16, G8, and G20 NH protons were observed even at low R = [drug/DNA] = 0.25 (Figure 2a,b) (Tables S1 and S2). These imino protons belong to the 3′-end tetrad. For the R ratio ranging from 0.5 to 1.0, the above-mentioned NH signals sharpened again in aloe emodin-8-glucoside complex (Figure 2b). Conversely, the signals remained broad in the aloe emodin complex and a yellow precipitate was observed at R = 1.0, preventing us to continue the titration (Figure 2a). The broadening suggested that these NH imino protons experienced a different molecular environment, most likely due to the mobility of the ligand inside the binging site (see the molecular modeling section). Furthermore, in aloe emodin spectra some minor signals were detected between 11.2 and 10.6 ppm, suggesting the formation of new G-quadruplex species during the titration. By increasing the ratio R at 1.50, the spectrum of aloe emodin-8-glucoside complex did not change significantly in comparison with the spectrum at R = 1.0, but a slight line broadening was observed, most likely due to a decrease in transparency of the solution, which turned opaque from clear (Figure 2b). At R = 2.0 significant changes in the spectrum were observed within two hours, accompanied by the formation of a yellow precipitate. At this ratio some signals experienced a downfield shift because of the aggregation/precipitation processes. The NH signals of G4, G16, G8, and G20 became broad (Figure 2b).

**Figure 3:**
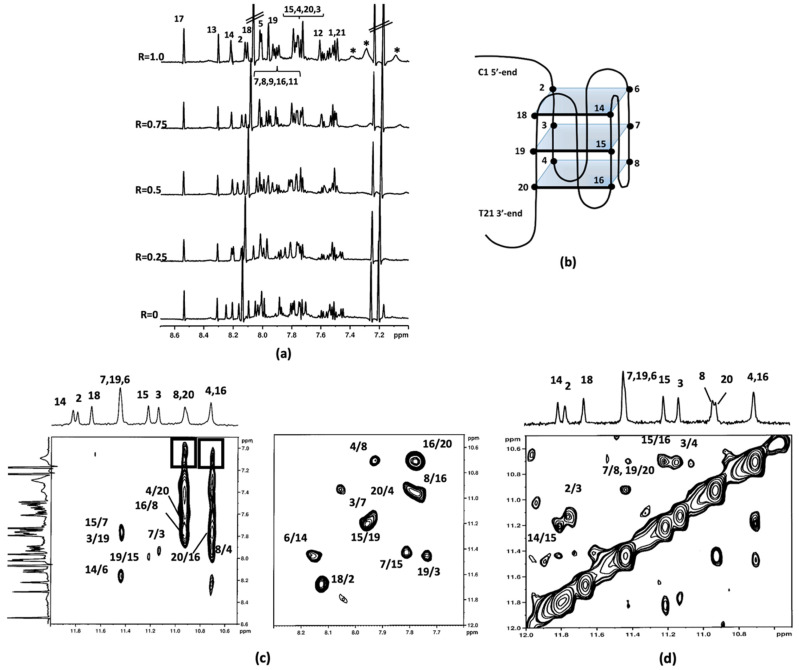
(**a**) Aromatic protons region of the ^1^H NMR titration spectra of c-kit21T12T21 with aloe emodin-8-glucoside (**2**). The numbers indicate the assignation to the corresponding imino protons, the dotted lines highlight the change in chemical shifts. The asterisks (*) mark the aromatic protons of **2**. (**b**) Schematic representation of the G-quadruplex structure. (**c**,**d**) imino-aromatic and imino-imino regions of 2D NOESY spectra (τm = 350 ms) of c-kit21T12T21 with **2**. Spectra were acquired at 25 °C in 90% H_2_O and 10% D_2_O, 20 mM KCl, and 5 mM K-phosphate buffer, pH 6.9, R = 2.0.

In the case of aloe emodin-8-glucoside (**2**) complex, at R = 1.0 broad signals were observed in the spectral region ranging from 7.4 to 7.0 ppm, attributable to the ligand aromatic protons, even if their unambiguous assignment became impossible (Figure 3). By increasing the R ratio up to 2, the signals of **2** became very broad and low in intensity.

To implement the information provided by the 1D NMR studies, 2D NOESY experiments were carried out at different R ratios. Figure 3 depicts imino-imino and imino-aromatic regions of 2D NOESY for **2**/c-kit21T12T21 complex at R = 2.0. The shift variation of the guanine imino protons gave the first evidence of the location of the ligands. The imino peaks corresponding to the guanines of the 3′-end G-quartet (G4-G8-G16-G20) were the most shifted. The chemical shift variation Δδ was in the range of −0.18/−0.36 ppm and −0.44/−0.32 ppm for aloe emodin and aloe emodin-8-glucoside complexes, respectively. These data confirmed that the most favorable binding site for both ligands is located at the 3′-end G-quartet of the quadruplex, which corresponds to an interaction at the 3′-face. The middle (G3-G7-G15-G19) and the 5′-end tetrads (G2-G6-G14-G18) were less affected. Unfortunately, in aloe emodin (**1**)/c-kit21T12T21 complex spectra, the signals of the ligand were not clearly detectable, because of their broadening. This could be due to an intermediate exchange process between various possible poses within the 3′-binding site and no NOEs interactions were detected. Even for the aloe emodin-8-glucoside (**2**)/c-kit21T12T21 complex, only few and ambiguously assigned NOEs were available for NMR structure determination: NH imino protons of G8/G20 and G16/G4 showed NOEs with aromatic protons of the ligand at 7.05 ppm (Figure 3). The downfield chemical shift variation of Me and H6 of the T21 residue suggested that T21 itself was pushed away from the 3′-end tetrad because of the ligand interaction. Consequently, this residue was not stacked with G20, as in the free G-quadruplex. A de-shielding variation was also observed for H6C9, indicating that it was not stacked with C5 unit, as occurs in the free oligonucleotide. At 5′-end tetrad the base pairs formed by C1 and A13 residues in the free G-quadruplex structure made this tetrad less accessible as a binding site.

#### 2.1.2. Interaction of Compounds **1**–**3** with Pu22T14T23

To confirm the preferential interaction of compounds **1**–**3** with G-quadruplex parallel topology, as demonstrated by Das and Dutta [24], the selected *Aloe vera* anthraquinone derivatives were tested on Pu22T14T23 oligonucleotide. 

**Figure 4:**
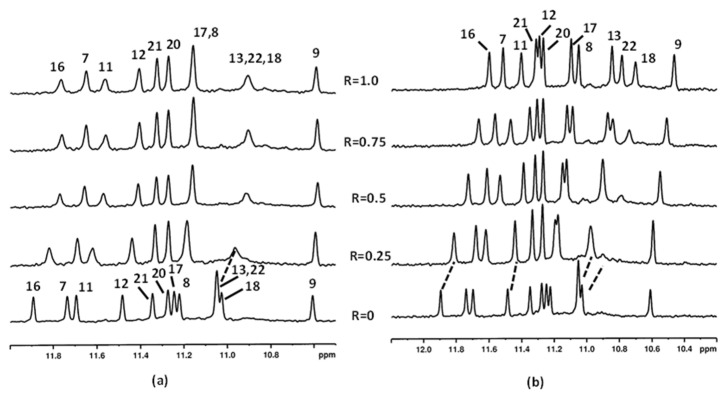
Imino protons region of the ^1^H NMR titration spectra of Pu22T14T23 with (**a**) aloe emodin (**1**) and (**b**) aloe emodin-8-glucoside (**2**).

Increasing amounts of aloe emodin (**1**) and aloe emodin-8-glucoside (**2**) were added to Pu22T14T23 solution. In both cases the formation of a precipitate at the ratio R ˃ 1.0 precluded the execution of the experiments at higher ratios. The NH imino protons remained in a region ranging from 12.0 to 10.5 ppm, indicating that the G-quadruplex structure was conserved after the interaction with the ligands. The NH signals in aloe emodin (**1**)/Pu22T14T23 complex remained quite sharp, with the exception of the imino protons belonging to 3′ (G13, G18 and G22) and 5′ (G11 and G16) quartets (Figure 4a). As regards the aloe emodin-8-glucoside (**2**)/Pu22T14T23 complex, all the NH imino signals appeared quite sharp (Figure 4b). A comparison of NH chemical shifts between the two complexes showed that aloe emodin-8-glucoside **2** induced greater chemical shift variations (G22 NH and G18 NH, Δδ −0.30/−0.34 ppm, G7, G11, G16NH, Δδ from −0.28 to −0.38 ppm) than aloe emodin **1** (Δδ ranging from −0.10 to −0.12 ppm) at the same ratio R = 1.0 (Tables S3 and S4). In both complexes, the chemical shift variations indicated that the ligand molecule can be located below 3′ or above 5′-end, as schematically reported in Figure 3d). The stoichiometry of 1:1 reached in the titration experiments could be explained with an internal competition of two binding sites at 3′- and 5′-ends in a fast exchange regime.

**Figure 5:**
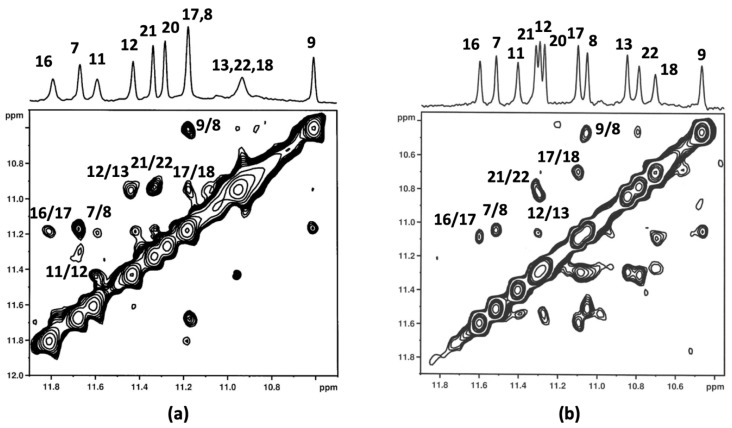
Imino protons region of 2D-NOESY spectra of Pu22T14T23 G-quadruplex with (**a**) aloe emodin **1** and (**b**) aloe emodin-8-glucoside **2** at 25 °C in H_2_O/D_2_O (9:1), 70 mM KCl, 25 mM potassium phosphate buffer (pH 6.9) and R = 1.0.

In both complexes the assignments were performed starting from the upfield signal of G9 NH, which remained constant during the titration experiments. The G8 NH and G7 NH could be attributed through their sequential NOEs, as well as G16 NH, which was easily attributed, allowing in this way to assign the signal of G17 NH. Signals of G21 NH and G20 NH showed small chemical shift changes in comparison with the free oligonucleotide and allowed to identify G22 NH in both complexes. The last upfield signal was attributed to G18 NH based on the sequential NOE with G17 NH (Figure 5).

**Figure 6:**
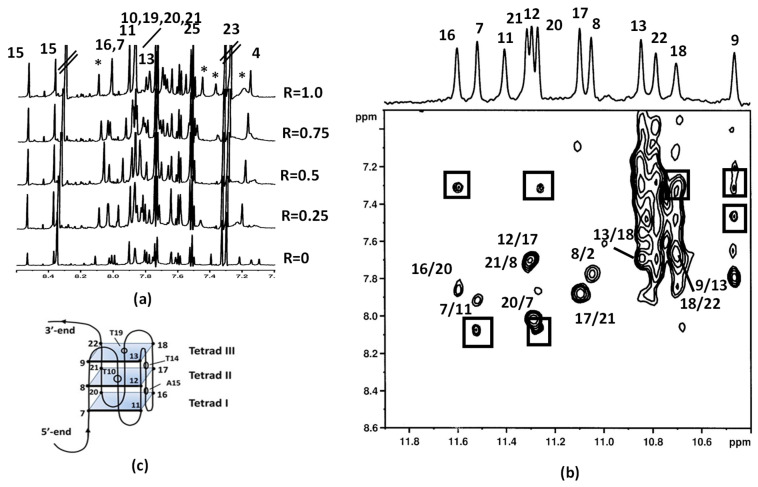
(**a**) Aromatic protons region of the ^1^H NMR titration spectra of Pu22T14T23 with aloe emodin-8-glucoside. Aromatic protons of **2** are indicated by asterisk (**b**) 2D NOESY spectrum of Aloe emodin-8-glucoside at 25 °C in H_2_O/D_2_O (9:1), 70 mM KCl, 25 mM potassium phosphate buffer (pH 6.9), R = 1.0. The boxes indicate the intermolecular NOE interactions between imino protons of the G-quadruplex and aromatic protons of the ligand. (**c**) Schematic representation of G-quadruplex structure.

In the complex aloe emodin-8-glucoside (**2**)/Pu22T14T23, G11 NH at 11.32 ppm interrupted the sequential interactions, lacking the NOE G11 NH–G12 NH. On the contrary, in aloe emodin (**1**)/Pu22T14T23 complex, this signal could be easily assigned and, consequently, G12 NH and G13 NH could be attributed. The G13 NH in the aloe emodin-8-glucoside complex allowed to attribute G12 NH (Figure 6). In the aromatic protons assignment, only few signals could be followed. The remaining attributions were performed by the inter-residue interactions with the NH imino protons (Tables S3 and S4).

Aloe emodin **1** aromatic protons were not detected, lying in a very crowded region overlapped with the aromatic protons of the oligonucleotide. Conversely, the aromatic protons of **2** were detected at 8.10, 7.47, 7.38, and 7.21 ppm (Figure 6a) and NOE contacts of G9 NH with the aromatic protons of the ligand at 7.47 and 7.38 ppm, were identified. Specifically, protons at 7.47, 7.38 and 7.21 were found to belong to ring A, according to TOCSY experiments. Other NOE interactions at the level of the 3′-end were found to connect G18 to the aromatic protons of the ligand, confirming the position of the ligand on top of the tetrad III, over the G9 and G18 bases. At the 5′-end, NOE contacts of the protons of tetrad I G20 and G7 NH with the aromatic proton of the ligand were observed. Unfortunately, no contacts were detected between the sugar moiety of aloe emodin-8-glucoside **2** and DNA, due to the overlapping between the sugar protons and the ribose protons of G-quadruplex (Figure 6b,c).

**Figure 7:**
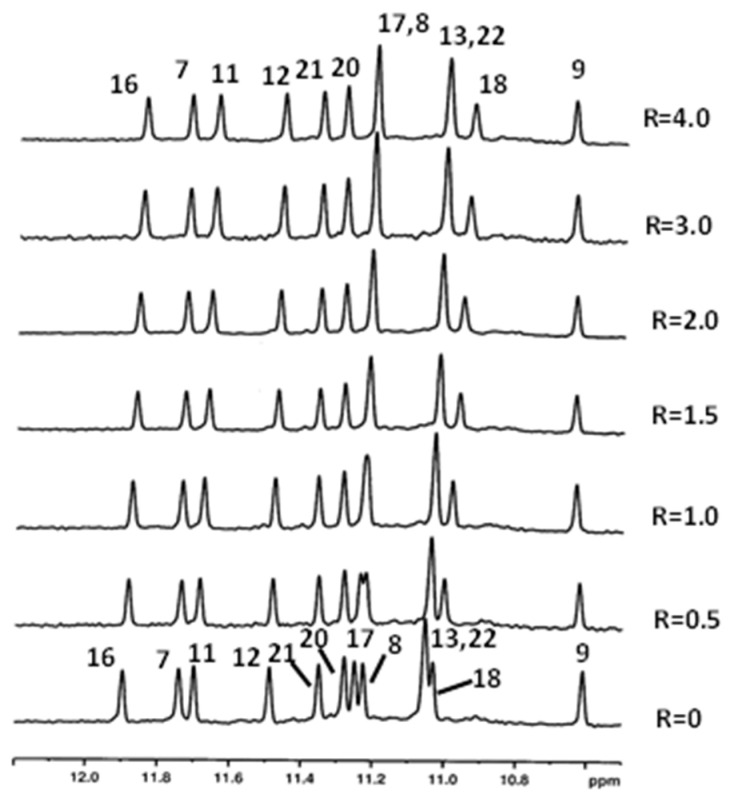
Imino protons region of the ^1^H NMR titration spectra of Pu22T14T23 with aloin **3** at different R = [aloin]/[Pu22T14T23] ratios.

Notwithstanding the unpromising results obtained by Dutta by investigating aloin **3** [24], NMR titration experiments were carried out also with this compound, to verify the coherence of the followed approaches. The titration was performed by adding aloin **3** to the Pu22T14T23 solution till ratio R = [drug]/[DNA] = 4.0 because of its higher solubility, compared to compounds **1** and **2**. Despite the high ratio, the NH imino protons signals, as well as the aromatic and methyl proton signals, remained sharp and only very small chemical shift variations were detected (Figure 7 and Table S5). Additionally, no NOE interactions were found between aloin **3** and Pu22T14T23. Overall, the results confirmed that the position of the sugar moiety is crucial for the binding to G-quadruplex structure.

#### 2.1.3. Molecular Modeling Studies

The aloe emodin (**1**) anthraquinone scaffold is located on the G4-G8-G16-G20 tetrad at the 3′-end of the *C-Kit* G-quadruplex sequence. The A ring of aloe emodin points outward and interacts with the aromatic system of C5 through a π-π stacking interaction. The B ring lies above the G4 and G8 nucleotides and is capable of forming two pairs of π-π stacking interactions with their π-systems. The C ring is located near the center of the G4-G8-G16-G20 tetrad and is engaged in four π-π stacking interactions with the aromatic systems of the G4 and G8 bases. Two hydrogen bonds are formed between the 8-OH group and C9OP1 (2.15 Å), and between the CH_2_OH group and G20O6 (2.62 Å). Furthermore, an intramolecular hydrogen bond between 1-OH and carbonyl group (2.03 Å) should be noted.

**Figure 8:**
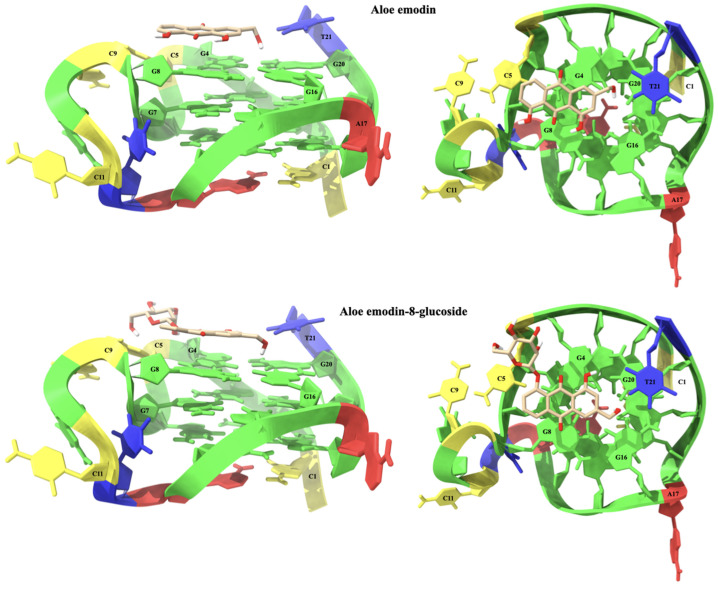
Lateral (**left**) and top (**right**) views of the aloe emodin (**1**) and aloe emodin-8-glucoside (**2**) complexes with the *C-Kit* G-quadruplex sequence. The nucleotides are shown as slabs and filled sugars: Adenine in red, cytosine in yellow, guanine in green, and thymine in blue. Labels are added to highlight key nucleotides. The ligand molecules are rendered in sticks and colored according to the atoms and only polar hydrogens are shown.

Aloe emodin-8-glucoside (**2**) is placed in a similar way to its aglycone **1**, but the presence of the 8-glucoside group leads to a rotation of the anthraquinone system. The A ring of the glucoside still points outward from the system, but in this case forming a π-π stacking interaction with G8 instead of C5. The central ring B is still positioned above the G4 and G8 nucleotides pair, but is shifted towards G8. This leads to two π-π stacking interactions with G8 but only one with the π-system of G4. As a result of this shift of the anthraquinone system, the C ring moves away from G8 and is able to form a unique π-π stacking interaction with the π-system of G4. Aloe emodin-8-glucoside interacts with *C-Kit* by means of an extensive network of hydrogen bond interactions that concours to stabilize the complex. The CH_2_OH group makes hydrogen bonds with T21H3 (2.22 Å), G20O6 (2.08 Å) and G16O6 (2.76 Å). The glucoside unit in turn interacts with G4 and C5. In particular, two hydrogen bonds, the first with 2-OH (2.16 Å) and the second between 3-OH (2.14 Å) are established with G4O3′, while nucleotide C5 is involved in two other hydrogen bonds, one between 4-OH and C5OP2 (2.75 Å) and one between CH_2_OH and C5O4’ (2.47 Å). The structures of the complexes of aloe emodin and aloe emodin-8-glucoside with *C-Kit* are shown in Figure 8.

**Figure 9:**
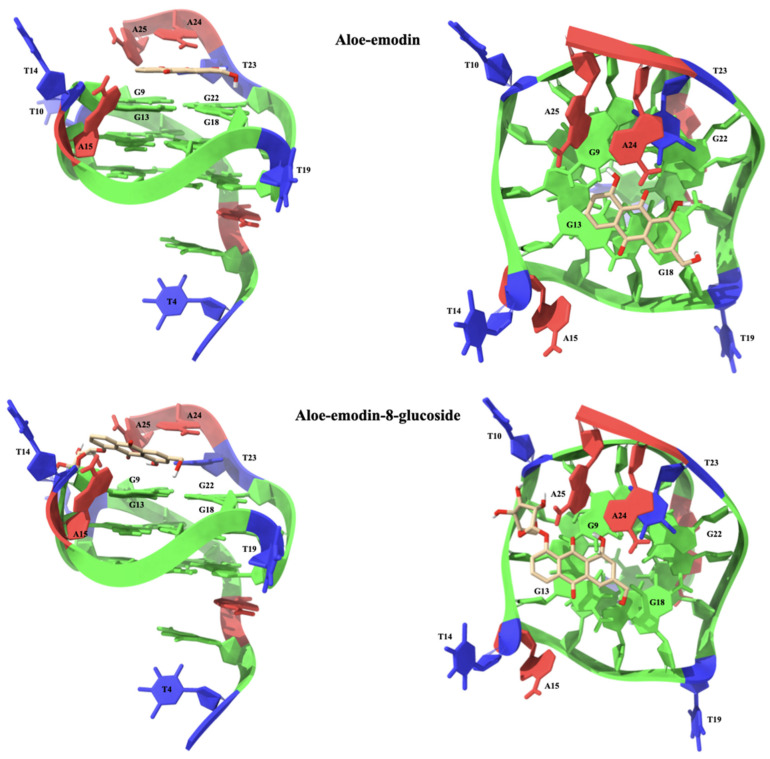
Lateral (**left**) and top (**right**) views of the aloe emodin (**1**) and aloe emodin-8-glucoside (**2**) complexes with the *c-Myc* G-quadruplex sequence. The nucleotides are shown as slabs and filled sugars: adenine in red, cytosine in yellow, guanine in green, and thymine in blue. Labels are added to highlight key nucleotides. The ligand molecules are rendered in sticks and colored according to the atoms and only polar hydrogens are shown.

Aloe emodin (**1**) interacts with *c-Myc* by placing itself above the G9-G13-G18-G22 tetrad at 3′-end. The A ring of the anthraquinone system is shifted towards G13, leading to the formation of two π-π stacking interactions with the nucleotide aromatic system. In this conformation, the central ring B is located between the bases G13 and G18, forming three π-π stacking interactions, one with the aromatic system of G13 and two with G18. In turn, the C ring is located above G18, leading to an additional π-π stacking interaction. Finally, the nucleotide A24 makes two hydrogen bonds with 8-OH (2.63 Å) and the carbonyl group (2.30 Å). Due to the effect of the glucoside group, the anthraquinone system of aloe emodin-8-glucoside undergoes a rotation that forces it to move away from the center of the G9-G13-G18-G22 tetrad. Ring A shifts outward and loses any kind of interactions with the tetrad. The central ring lies above G13 and is therefore capable of interacting with the nucleoside via two π-π stacking interactions. Ring C is located between the π-systems of G9, G13, and A24, with which it interacts through four π-π stacking interactions. The binding conformation is stabilized by an extensive network of hydrogen bonds. The CH_2_OH group makes two hydrogen bonds with G18N7 (2.81 Å) and G18O6 (2.68 Å), while 1-OH makes an intramolecular hydrogen bond with the carbonyl group (2.02 Å). The anomeric oxygen gives rise to a hydrogen bond with A25H61 (2.49 Å). The structure of the complex is further stabilized by two hydrogen bonds between T10OP2 and the hydroxyl groups of 2-OH (2.58 Å) and 3-OH (1.79 Å). The structures of the complexes of *c-Myc* with aloe emodin and aloe emodin-8-glucoside are shown in Figure 9.

**Figure 10:**
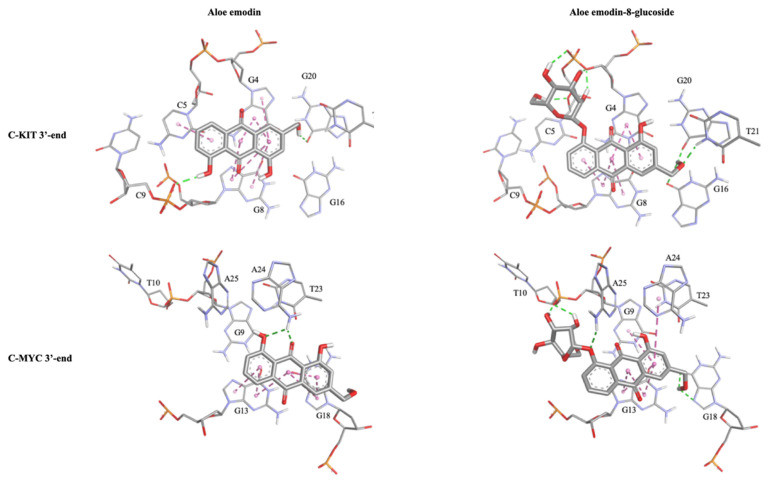
Detailed representations of the *C-Kit* and *c-Myc* binding sites, composed of the ligands (rendered in sticks) and key nucleotides (rendered in thick sticks and labeled). Non-bonded interactions were represented as dotted lines. Molecular graphics and analyses were performed using BIOVIA Dassault Systémes Discovery Studio Visualizer (DSV2021).

For both G-quadruplex sequences (*C-Kit* and *c-Myc*) the results of the molecular modeling studies confirmed the privileged interaction of aloe emodin and aloe emodin-8-glucoside with the 3′-end tetrads. The complexes thus obtained are stabilized by an extensive network of interactions, both π-π stacking and hydrogen bond. The observed differences in the binding modes of aloe emodin and aloe emodin-8-glucoside with the *C-Kit* and *c-Myc* models are congruent with those expected. The presence of the β-glucose moiety in fact allows for a more efficient interaction with the G-quadruplexes, without however compromising their structural integrity. The details of the key interactions of the four complexes are shown in Table 1 and Figure 10.

Molecular modeling studies on the complex aloin/G-quadruplex were not performed, as the data from NMR titration confirmed the lack of interactions with both the G-quadruplex structures.

## 3. Materials and Methods

### 3.1. Ligands

Aloin was purchased from Xi’an Haoxuan Bio-Tech Co., Xi’an, China. The pure compound (0.95 g, 48%) was obtained after two recrystallization steps of the commercial aloin (2 g) from methanol (2 mL). Lemon-yellow needles, m. p. 148 °C. TLC: R*f* value = 0.46 on silica gel (chloroform: methanol 4:1).

^1^H-NMR (600 MHz, MeOH-*d*_4_) δ: 7.47(1H, t, *J* = 8.0 Hz, H-6), 7.05 (1H, s, H-4), 7.02 (1H, d, *J* = 8.8 Hz, H-5), 6.86 (1H, s, H-2), 6.83 (1H, t, *J* = 8.0, H-7), 4.64 (2H, d, *J* = 3.6 Hz, 3-CH_2_-OH), 4.56 (1H, s, H-10), 3.38 (1H, dd, *J* = 9.2, 2.0 Hz, H-1′), 3.01 (1H, t, *J* = 9.2, H-2′), 3.23 (1H, t, *J* = 8.8 Hz, H-3′), 2.89 (1H, t, *J* = 8.8 Hz, H-4′), 2.91 (1H, m, H-5′), 3.54 (1H, dd, *J* = 1.6, 11.6 Hz, H-6′), 3.40 (1H, dd, *J* = 4.0, 9.6 Hz, H-6). ^1^H NMR spectrum of aloin was dissolved in 90% H_2_O and 10% D_2_O, 20 mM KCl, and 5 mM K-phosphate buffer, and pH 6.9, as shown in Figure S1.

Aloe-emodin was obtained from aloin by an oxidation reaction with ferric chloride as reported by Hay [33]. Briefly, a solution of aloin (**3**) (1.00 g, 2.39 mmol) and ferric chloride hexahydrate (3.10 mmol) in water (15 mL) was heated under reflux for 15 min and then at 125 °C for 6 h. The solution was cooled and the solid was collected, dried, and extracted with boiling toluene. Removal of toluene yielded a crude compound, which upon recrystallization from ethanol gave aloe-emodin (**1**) as a reddish-orange solid (0.58 g, 58%). ^1^H-NMR (600 MHz, DMSO-*d*_6_) δ: 11.95 (1H, s, C8-OH), 11.89 (1H, s, C1-OH), 7.78 (1H, H-6), 7.68 (1H, H-5), 7.66 (1H, H-4), 7.34 (1H, H-7), and 7.25 (1H, H-2).

Aloe-emodin-8-glucoside (**2**) was purchased from Merck. ^1^H-NMR (600 MHz, Pyridine-*d*_5_) δ: 13.34 (1H, br, -OH), 8.11 (1H, brs, H-4), 8.04 (1H, d, *J* = 8.0 Hz, H-5), 7.99 (1H, d, *J* = 8.0 Hz, H-7), 7.67(1H, brs, H-2), 7.60 (1H, t, *J* = 8.0 Hz, H-6), 5.00 (3-CH_2_OH), 5.81 (1H, d, *J* = 7.5 Hz, H-1′), 4.60 (1H, dd, *J* = 12.0 Hz, *J* = 2.5 Hz, H-6′a), 4.57 (1H, t, *J* = 8.5 Hz, H-2′), 4.43 (1H, t, *J* = 7.5 Hz, H-4′), 4.42 (1H, dd, *J* = 12.5 Hz, *J* = 5.5 Hz, H-6′b), 4.38 (1H, t, *J* = 8.5 Hz, H-3′), 4.25 (1H, m, H-5′). ^1^H NMR spectrum of aloe-emodin-8-glucoside was dissolved in 90% H_2_O and 10% D_2_O, 20 mM KCl, and 5 mM K-phosphate buffer, and pH 6.9, as shown in Figure S2.

### 3.2. NMR Experiments

The quadruplex NMR samples were prepared at a concentration of 0.4 mM (in 0.6 mL (H_2_O/D_2_O 9:1) buffer solution having 70 mM KCl and 25 mM potassium phosphate buffer for Pu22T14T23 and 5 mM phosphate buffer and 20 mM KCl for c-kit21T12T21 (pH 6.9). NMR spectra were recorded with Bruker AV 600 MHz spectrometer. ^1^H chemical shifts were referenced relative to external sodium 2,2-dimethyl-2-silapentane-5-sulfonate (DSS). Monodimensional proton spectra were recorded using pulsed-field gradient for H_2_O suppression. Stock solution of the ligands were prepared in DMSO-*d*_6_. ^1^H NMR titrations were performed at 25 °C by adding increasing amounts of ligand to the DNA at different ratios: R = [drug]/[DNA]. The protons in the complexes were assigned by using NOESY and TOCSY experiments. Phase sensitive NOESY spectra were acquired at 25 °C, in TPPI mode, with 2048 × 1024 complex FIDs. Mixing times ranged from 200 ms to 350 ms. TOCSY spectra were acquired with the use of a MLEV-17 spin-lock pulse (60 ms total duration). All spectra were transformed and weighted with a 90° shifted sine-bell squared function to 4K × 4K real data points. NOESY and TOCSY spectra were analyzed for solution with R = [drug]/[DNA] = 1.0, 2.0 or 4.0.

### 3.3. Molecular Modeling Studies

The NMR ensemble models deposited in the Protein Data Bank for *C-Kit* (PDB accession code: 2KYP) [30] and *c-Myc* (PDB accession code: 2L7V) [28] were used to obtain the starting 3D-structures.

Molecular docking calculations for **1** and **2** were performed by AutoDock 4.2 [34,35], using the LGA (Lamarckian Genetic Algorithm) together with a grid-based energy evaluation method to calculate grid maps, using a 80 Å × 80 Å × 80 Å box with a spacing of 0.01 Å. The phosphorus atoms in the DNA were parameterized using the Cornell parameters. The AutoDock Toolkit (ADT) [36] was used to add the Gasteiger–Marsili charges [37] to the ligands and the Addsol utility of AutoDock was used to set up the solvation parameters. The initial population for each molecule consisted of 100 randomly placed individuals, with a maximum number of 250 energy evaluations and an elitism value of 1, a mutation rate of 0.02, and a crossover rate of 0.80. The local search was conducted by applying the so-called pseudo-Solis and Wets algorithm with a maximum of 250 iterations per local search and 250 independent docking runs. The docking results were scored by using an in-house version of the simpler intermolecular energy function based on the Weiner force field, and the lowest energy conformations (differing by less than 1.0 Å in positional root-mean-square deviation (rmsd)) were collected.

The resulting complexes were placed at the center of a box (boundaries at 2.0 nm apart from all atoms) and solvated with TIP3P water molecules [38]. Amber ff99 force field [39] with bsc1 corrections [40] was used to describe the *C-Kit* and *c-Myc* G-quadruplexes. To remove bad contacts, 1000 minimization steps were performed on the initial systems, followed by a heating ramp of short (100 ps) consecutive simulations. The production simulations consisted of 5 ns of Langevin Molecular Dynamics (LMD) [41,42] NPT equilibration at 298 K and 1 atm, as implemented in NAMD [43]. During this step, all bonds to hydrogen atoms were constrained using the SHAKE algorithm [44]. Water molecules were kept rigid with SETTLE [45], allowing an integration time step of 0.002 ps. The electrostatic interactions were calculated using the Particle Mesh Ewald (PME) method (Coulomb cut-off radius of 1.2 nm) [46,47]. A Berendsen thermostat (coupling time of 0.1 ps) was applied to the systems [48]. Molecular graphics and analyses were performed with UCSF ChimeraX, developed by the Resource for Biocomputing, Visualization, and Informatics at the University of California, San Francisco, with support from National Institutes of Health R01-GM129325 and the Office of Cyber Infrastructure and Computational Biology, National Institute of Allergy and Infectious Diseases [49,50].

## 4. Conclusions

*Aloe vera* derived natural compounds aloe emodin (**1**), aloe emodin-8-glucoside (**2**), and aloin (**3**) have recently attracted great interest for their promising antitumor activity. However, despite the well-known use of *Aloe vera* as a medicinal plant, studies on the molecular mechanism of activity of the single compounds are still scarce. Recent binding studies of *Aloe*-active compounds with G-quadruplex sequences identified aloe emodin **1** and aloe emodin-8-glucoside **2** as potent quadruplex binding molecules, mainly *C-Kit* and *c-Myc* sequences, whereas aloin **3** was found to weekly interact with the nucleotides. We investigated the interaction between the same anthraquinone derivatives and *c-Myc* and *C-Kit* G-quadruplex DNA structures by a different approach, based on NMR spectroscopy. Molecular modeling calculations supported the NMR results and allowed us to propose the structures of *C-Kit*/ligands and *c-Myc*/ligands complexes. The obtained evidence confirmed the crucial role of the planar anthraquinone moiety of aloe emodin-8-glucoside and aloe-emodin, enabling them to bind the G-quadruplex structures. Unfortunately, the low solubility of compound **1** in aqueous solution hampered the investigation of the interactions with both G-quadruplex sequences at higher ligand concentrations. Overall, the obtained results confirmed the findings reported by Das and Dutta, pointing out the complementarity of the approaches. Furthermore, the construction of models of interaction of compounds **1** and **2** with G-quadruplex DNA structures could pave the way to the design of new nature-derived compounds endowed with increased G-quadruplex stabilizing activity.

## Figures and Tables

**Table 1:** Table summarizing the key interactions observed in the *C-Kit*/Ligands and *c-Myc*/Ligands complexes as obtained by molecular modeling. The table shows the nucleotides involved in the interactions together with the type of interactions (HB = Hydrogen Bond).

Ligands	*C-Kit* 3′-End Tetrad						*c-Myc* 3′-End Tetrad						
	G4	C5	G8	C9	G16	G20	G21	G9	T10	G13	G18	A24	A25
aloe emodin (**1**)	π-π	π-π	π-π	HB		HB				π-π	π-π	HB	
aloe emodin-8-glucoside (**2**)	HB, π-π	HB	π-π		HB	HB	HB	π-π	HB	π-π	HB	π-π	HB

## Data Availability

Not applicable.

## Supplementary Materials

The following supporting information can be downloaded at: https://www.mdpi.com/article/10.3390/ijms232416018/s1.
