# Supplementary material for: Investigation of the Interaction between Aloe vera Anthraquinone Metabolites and c-Myc and C-Kit G-Quadruplex DNA Structures
Source: Int J Mol Sci. 2022 Dec 16;23(24):16018. doi: 10.3390/ijms232416018 (PMC9788642; doi:10.3390/ijms232416018)
Supplement: Supplementary file 1 [file ijms-23-16018-s001.zip › ijms-2060910-supplementary.pdf]

Investigation of the Interaction between *Aloe vera* Anthraquinone Metabolites and *c-Myc* and *C-Kit* G-Quadruplex DNA Structures

Sabrina Dallavalle, Roberto Artali, Salvatore Princiotto, Loana Musso, Gigliola Borgonovo and Stefania Mazzini

Table S1. Selected ^{1}H chemical shift values of G-quartets for the complex of aloe emodin with c-kit21T12T21. S1

Table S2. Selected ^{1}H chemical shift values for the complex of aloe emodin-8-glucoside with c-kit21T12T21. S2

Table S3. Selected ^{1}H NMR chemical shift values for the complex of aloe emodin with Pu22T14T23 G-quadruplex. S3

Table S4. Selected ^{1}H NMR chemical shift values for the complex of aloe emodin-8-glucoside with Pu22T14T23 G-quadruplex. S4

Table S5. ^{1}H NMR chemical shift values of G quartets for the complex of aloin with Pu22T14T23 G-quadruplex. S5

Figure S1. ^{1}H NMR spectrum of aloin at 25 °C in 90% H_2O and 10% D_2O, 20 mM KCl, and 5 mM K-phosphate buffer, pH 6.9. S6

Figure S2. Selected region of ^{1}H NMR spectrum of aloe-emodin 8-glucoside at 25 °C in 90% H_2O and 10% D_2O, 20 mM KCl, and 5 mM K-phosphate buffer, pH 6.9. S6

Table S1. Selected ^{1}H chemical shift values of G quartets for the complex of aloe emodin with c-kit21T12T21.[a,]

c-kit21T12T21	H1/H2/H5/CH_3	$\Delta\delta^b$	H6/H8	$\Delta\delta^b$
C1	5.66	+0.02	7.48	−0.08
G2	11.89	−0.09	8.21	0.00
G3	11.21	−0.09	7.78	+0.03
G4	10.86	**−0.29**	7.76	+0.07
C5	6.17	−0.01	8.02	−0.03
G6	11.50	−0.02	8.05	+0.02
G7	11.50	−0.08	8.00	−0.02
G8	10.95	**−0.36**	n.d.	−0.05
C9	6.10	−0.04	7.59	−0.13
G10	n.d.	-	n.d.	-
C11	6.05	0.00	7.88	−0.08
T12	1.89	+0.01	7.56	−0.14
A13	n.d.	-	8.18	+0.01
G14	11.92	−0.02	8.21	−0.04
G15	11.29	−0.09	7.82	0.00
G16	10.86	**−0.18**	7.79	+0.03
A17	n.d.	-	8.54	+0.03
G18	11.74	−0.08	8.15	+0.07
G19	11.50	−0.06	7.99	−0.01
G20	10.97	**−0.28**	7.76	+0.09
T21	n.d.	-	n.d.	-

[a] Measured at 25 °C in ppm (δ) from external DSS. Solvent H_2O-D_2O (90:10 v/v), 5 mM phosphate buffer, 20 mM KCl, pH 6.9, R = 1.0. $^{b}\Delta\delta = \delta_{bound} - \delta_{free}$

Table S2. Selected ^{1}H chemical shift values for the complex of aloe emodin-8-glucoside with c-kit21T12T21.[a]

	R = [ligand]/[ckit21T12t21] = 1.0				R = [ligand]/[ckit21T12t21] =2.0			
c-kit	H1/H2/H5/CH_3	$\Delta\delta^b$	H6/H8	$\Delta\delta^b$	H1/H2/H5/CH_3	$\Delta\delta^b$	H6/H8	$\Delta\delta^b$
C1	5.64	0.00	7.50	−0.06	5.66	+0.02	7.47	−0.09
G2	11.76	**−0.20**	8.14	−0.03	11.91	**−0.10**	8.16	+0.01
G3	11.14	−0.16	7.75	0.00	11.25	−0.05	7.77	+0.02
G4	10.71	**−0.44**	7.80	+0.11	10.71	**−0.44**	7.80	+0.11
C5	6.18	0.00	8.02	−0.02	6.19	+0.01	8.02	−0.02
G6	11.45	−0.07	n.d.	-	11.53	+0.01	8.04	+0.02
G7	11.45	−0.13	7.94	−0.04	11.53	−0.05	8.00	+0.02
G8	10.95	**−0.36**	7.93	+0.15	10.95	**−0.36**	7.93	+0.15
C9	6.18	−0.05	7.82	**+0.10**	6.05	−0.08	7.88	**+0.16**
G10	n.d.	-	n.d.		n.d.	-	n.d.	-
C11	6.07	+0.02	7.93	−0.03	6.09	+0.04	7.95	−0.01
T12	1.89	−0.08	7.57	−0.13	1.80	−0.08	7.57	−0.13
A13	n.d.	-	8.31	**+0.15**	n.d.	-	8.31	**+0.15**
G14	11.82	−0.12	8.21	−0.10	11.95	−0.04	8.23	−0.02
G15	11.22	−0.16	7.82	0.00	11.33	−0.05	7.87	+0.05
G16	10.71	**−0.33**	7.77	+0.01	10.71	**−0.33**	7.77	+0.01
A17	n.d.	-	8.55	+0.04	n.d.	-	8.56	+0.05
G18	11.67	**−0.15**	n.d	-	11.77	−0.05	8.02	−0.06
G19	11.45	−0.11	7.96	−0.04	11.53	−0.03	8.03	+0.03
G20	10.93	**−0.32**	7.78	+0.11	10.93	**−0.32**	7.75	+0.08
T21	1.83	**+0.33**	7.51	**+0.36**	1.83	**+0.33**	7.51	**+0.36**

[a] Measured at 25 °C in ppm (δ) from external DSS. Solvent H_2O-D_2O (90:10 v/v), 5 mM phosphate buffer, 20 mM KCl, pH 6.9. [b]$\Delta\delta = \delta_{bound} - \delta_{free}$

Table S3. Selected ^{1}H NMR chemical shift values for the complex of aloe emodin with Pu22T14T23 G-quadruplex[a,b].

	H1/H2/Me	Δδ[c]	H6/H8	Δδ[c]
T4	1.57	−0.08	7.22	−0.02
G5	-	-	-	-
A6	-	-	-	-
G7	11.68	**−0.10**	8.03	+0.01
G8	11.18	−0.04	7.72	0.00
G9	10.60	0.00	7.78	+0.03
T10	2.04	+0.05	-	-
G11	11.60	**−0.11**	7.98	−0.01
G12	11.43	−0.07	7.88	−0.02
G13	10.95	**−0.11**	7.87	+0.01
T14	1.92	0.00	7.66	+0.01
A15	8.38	0.00	8.54	+0.01
G16	11.80	**−0.10**	8.09	−0.02
G17	11.19	−0.06	7.78	−0.02
G18	10.92	**−0.10**	7.72	−0.07
T19	2.04	+0.05	7.90	+0.04
G20	11.26	−0.02	-	-
G21	11.32	−0.05	7.88	−0.03
G22	10.92	**−0.12**	7.78	+0.17
T23	1.62	+0.14	7.27	+0.13
A24	-	-	-	-
A25	7.47	+0.08	7.57	+0.07

[a]Measured at 25 °C in H_2O/D_2O (9:1), 70 mM KCl, 25 mM potassium phosphate buffer (pH 6.9) and R = 1.0. [b]Measured in ppm from external DSS.
[c]$\Delta\delta = \delta_{bound} - \delta_{free}$.

Table S4. Selected ^{1}H NMR chemical shift values for the complex of aloe emodin-8-glucoside with Pu22T14T23 G-quadruplex[a,b].

	H1/H2/Me	$\Delta\delta^c$	H6/H8	$\Delta\delta^c$
T4	1.62	−0.04	7.15	−0.06
G5	-	-	-	-
A6	-	-	-	-
G7	11.46	**−0.28**	8.03	+0.01
G8	11.01	−0.21	7.72	0.00
G9	10.41	**−0.19**	7.78	+0.03
T10	2.01	+0.01	7.90	+0.04
G11	11.32	**−0.38**	7.90	−0.09
G12	11.27	−0.22	7.77	−0.02
G13	10.82	**−0.23**	7.79	+0.01
T14	1.92	0.00	7.66	+0.01
A15	8.37	0.00	8.52	+0.02
G16	11.53	**−0.37**	8.00	−0.02
G17	11.08	−0.16	7.70	−0.06
G18	10.67	**−0.34**	7.72	−0.07
T19	2.01	+0.01	7.90	+0.04
G20	11.27	−0.01	7.88	-
G21	11.27	−0.08	7.89	+0.07
G22	10.73	**−0.30**	7.68	+0.17
T23	1.42	−0.06	7.27	+0.13
A24	-	-	-	-
A25			7.50	0.00

[a]Measured at 25 °C in H_2O/D_2O (9:1), 70 mM KCl, 25 mM potassium phosphate buffer (pH 6.9) and R = 1.0. [b]Measured in ppm from external DSS.
[c]$\Delta\delta = \delta_{bound} - \delta_{free}$.

Table S5. ^{1}H NMR chemical shift values of G quartets for the complex of aloin with Pu22T14T23 G-quadruplex[a,b].

	H1/H2/Me	$\Delta\delta^{c}$
G7	11.69	−0.07
G8	11.20	−0.02
G9	10.62	+0.02
G11	11.60	−0.11
G12	11.42	−0.08
G13	10.95	−0.11
G16	11.80	−0.10
G17	11.20	−0.05
G18	10.96	−0.16
G20	11.18	0.00
G21	11.35	−0.02
G22	10.95	−0.09

[a]Measured at 25 °C in H_2O/D_2O (9:1), 70 mM KCl, 25 mM potassium phosphate buffer (pH 6.9) and R = 4.0. [b]Measured in ppm from external DSS.
[c]$\Delta\delta = \delta_{bound} - \delta_{free}$.

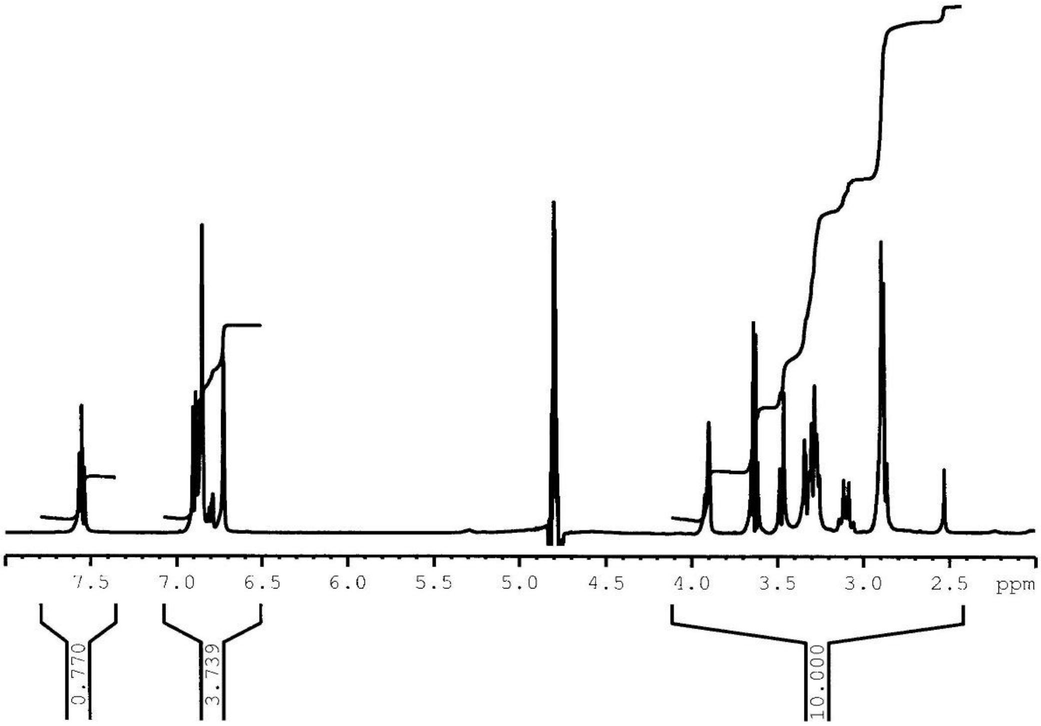

Figure S1. ^{1}H NMR spectrum of aloin at 25 °C in 90% H_2O and 10% D_2O, 20 mM KCl, and 5 mM K-phosphate buffer, pH 6.9.

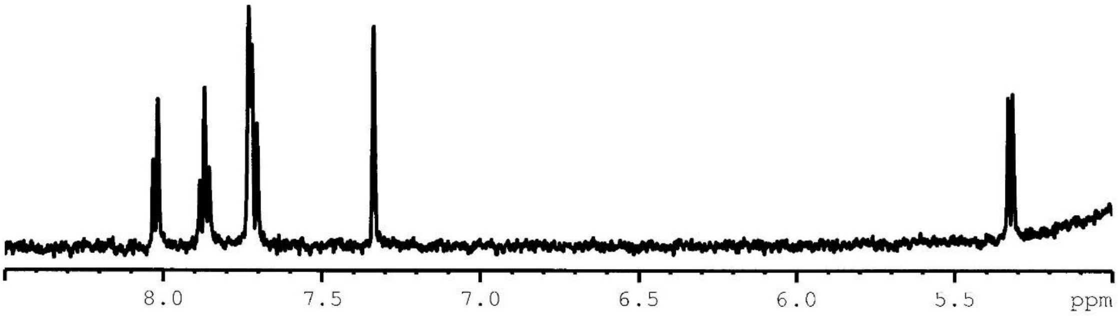

Figure S2. Selected region of ^{1}H NMR spectrum of aloe-emodin 8-glucoside at 25 °C in 90% H_2O and 10% D_2O, 20 mM KCl, and 5 mM K-phosphate buffer, pH 6.9.
